# The economic value of human milk from three cohort studies in Friuli Venezia Giulia, Italy

**DOI:** 10.1186/s13006-024-00618-z

**Published:** 2024-02-08

**Authors:** Benedetta Zabotti, Sara Buchini, Mariarosa Milinco, Adriano Cattaneo, Paola Pani, Luca Ronfani

**Affiliations:** 1 School of Nursing, Clinical Department of Medical, Surgical and Health Sciences, University of Trieste, Trieste, Italy; 2 Health Management Department, Institute for Maternal and Child Health, IRCSS “Burlo Garofolo”, Trieste, Italy; 3 Clinical Epidemiology and Public Health Research Unit, Institute for Maternal and Child Health IRCCS “Burlo Garofolo”, Via Dell’Istria 65/1, 34137 Trieste, Italy; 4 IBFAN Italia, Trieste, Italy

**Keywords:** Breastmilk, Breastfeeding, Monetary value, Italy

## Abstract

**Background:**

The Mothers’ Milk Tool, developed and launched by the Australian National University and Alive & Thrive in 2022, allows to estimate the volume and value of breastmilk using prevalence rates of breastfeeding by month of age from birth to 36 months. The objective of this study was to obtain these estimates for three cohort studies conducted in a region of Italy.

**Methods:**

Breastfeeding data from three cohort studies carried out in 1999, 2007 and 2016, with follow-up to 12, 24 and 36 months of 842, 400 and 265 children, respectively, were entered into the downloadable version of the tool. Breastfeeding rates charts and tables with estimates of breastmilk production and value for breastfeeding of children aged 0–36 months were produced.

**Results:**

The rates of initiation of breastfeeding were similar in the three cohorts, while the rates of any breastfeeding at subsequent ages increased over the years. The volumes and values of breastmilk per child increased accordingly, from around 130 L (13,000 USD) in 1999, to 200 L (20,000 USD) in 2007, to 226 L (22,600 USD) in 2016. The percentage of lost breastmilk decreased from 67.7% to 55.4% to 43.7%, respectively. Overall, the 1507 mothers of the three cohorts produced an estimated 250,000 L of breastmilk for their children aged 0–36 months. At 100 USD per litre, this would add up to around 25 million USD.

**Conclusions:**

Our study shows that the Mothers’ Milk Tool can be used to estimate per child volumes and values of breastmilk produced and lost at local levels, and to provide simple indicators of the effects of breastfeeding interventions using the percentage of lost breastmilk, where datasets on rates of breastfeeding by month of age are available. The results of such studies can be used to advocate for better and adequately funded programmes for the protection, promotion and support of breastfeeding.

## Introduction

The evidence on the benefits of breastfeeding or, better, the harms of not breastfeeding for the short and long term health of mothers and children is overwhelming [1, 2]. There is plenty of evidence also for the consequences of suboptimal breastfeeding for the health and social system, in terms of inequalities and of medical and non-medical costs [3]. It was estimated that in 2022, globally, breastfeeding had the potential to save 574 billion USD (0.7% of global Gross Domestic Product, GDP), and more than 10% of household’s wages by not having to purchase infant formula [4]. This estimate, however, fails to take into account the cost of additional unpaid household care for children; if taken up, this would push the costs of not breastfeeding even higher [5]. More recently, attention has been paid to the environmental impact of formula feeding and of the dairy and baby food industry [6–8]. The cost of formula feeding for the environment should be added to the total cost of not breastfeeding [9].

In contrast to the increasing attention paid to the cost of not breastfeeding, little attention has so far been given to the economic value of breastmilk, of breastfeeding and, more generally, of unpaid care work, mostly done by women. Back in 1988, feminist economists criticised the failure to include reproductive functions and breastfeeding in GDP [10]. In 1999, Smith estimated the amount of breastmilk produced in Australia [11]. Two years later, she made a concrete proposal for the inclusion of breastmilk production in the national accounting system. Using the figures estimated in 1999, she calculated that the net economic value of breastfeeding in Australia could have been set at around 2 billion AUD per year and the capitalised value at 37 billion AUD, i.e. far more than the value of Australia’s livestock (17.9 billion AUD) and plantation forests (4.5 billion AUD). She also estimated that if all Australian children were breastfed according to the WHO recommendations, the value of human milk production would increase by nearly three times, standing alongside Australia’s subsoil mineral assets [12].

Encouraged by the fact that three renowned economists, among them two Nobel Prize laureates, had cited breastmilk as suitable to include in GDP with important implications for national output measurement and policies [13], a research partnership between health economists and health professionals at the Australian National University and the Alive & Thrive Southeast Asia & Pacific developed the Mother’ Milk Tool (MMT). Previous tools had meanwhile been developed to estimate the cost of not breastfeeding and the investment needed to scale up the protection and promotion of breastfeeding [14, 15]. The downloadable MMT was made freely available to potential users [16] and a paper was published to explain how it was developed and how it can be used, including a discussion of its current limitations [17]. Among these, according to the authors, the fact that breastfeeding prevalence data, essential to yield precise results, are particularly lacking in high-income countries. For this reason, the MMT has a built-in prediction model that allows to bridge data gaps.

The only national data on the prevalence of breastfeeding at different ages in Italy were published in 2013 [18]. Beyond being old, these data refer to four relatively wide age periods: 0–1, 2–3, 4–5 and 6–12 months, with no data for the second and third year of life. The MMT has a built-in predictor that helps generate monthly rates of breastfeeding from birth to three years of age based on at least three available data points. However, the precision of the results, in terms of volume and value of breastmilk, increases with an increasing number of data points. Contrary to most regions in Italy, Friuli Venezia Giulia (FVG), a region in the north-east with 1.2 million people, has good and reliable data on breastfeeding. Between 2005 and 2020, the rates of exclusive breastfeeding at hospital discharge and at 4–6 months showed encouraging improvements in FVG, but are still far from desirable [19]. To add arguments for further improvements, the objective of this study was to estimate the volume and monetary value of human milk produced by three cohorts of mothers in different years, and to test the usefulness of the MMT for evaluating regional level changes over time.

## Methods

We started by downloading the MMT and by getting familiar with its use. To do so, we ran several of the pre-loaded databases from different countries worldwide and we learned how to input data using, at the beginning, series of mock breastfeeding rates. We then retrieved and used real data from three cohort studies. Table 1 shows the main features of these studies.

**Table 1:** Main features of the three cohort studies

Main features	Cohort 1 (1999)	Cohort 2 (2007)	Cohort 3 (2016)
Sample size	842	400	265
Inclusion criteria	Healthy term newborns	Healthy term newborns	All infants (no preterm < 30 weeks)
Recruitment site	Hospital	Hospital	Paediatric practice
Recruitment time	Soon after birth	Soon after birth	At 1–4 weeks of age
Follow up	12 months	24 months	36 months
Assessments	At birth and every month up to 12 months of age	At birth and at 1, 3, 6, 9, 12 18 and 24 months of age	At birth and at 1, 3, 5, 8, 12, 18, 24 and 36 months of age
No or invalid data (lower rate)	1% at 1 month	28% at 1 month	5% at 1 month
No or invalid data (higher rate)	7% at 11 months	67% at 24 months	15% at 36 months

The first cohort study was carried out to compare the cost of health care between breastfed and non-breastfed infants [20]. Out of 9,100 live births recorded in 1999 in FVG, a total of 842 mothers with their healthy newborn infants complying with pre-established inclusion criteria were enrolled soon after hospital birth between January and August 1999, and were followed up monthly to age 12 months. The denominators for the computation of the rates of any breastfeeding were always lower than 842 due to missing or invalid data. However, the loss to follow up was generally slight; the lower rate of attrition was 1% at one month of age, and the higher was 7% at 11 months.

The second cohort of 400 mothers with their healthy newborn infants was enrolled between July 2007 and July 2008 (10,569 and 10,515 annual live births, respectively, in FVG) and was followed up to 24 months of age, with data on breastfeeding gathered at 1, 3, 6, 9, 12, 18 and 24 months [21, 22]. This was actually a sub-cohort of a larger one intended to study the effect of exposure to heavy metals during pregnancy on the neuro- and psychological development of their offspring [23]. This large study was very demanding, also because of the need to collect several biological samples, and many women refused to participate or withdrew after some time [24]. As a consequence, the loss to follow up was considerable, with data on breastfeeding available from 287 (72%) of mothers at one month, 235 (59%) at 6 months, 165 (41%) at 12 months, and only 132 (33%) at 24 months.

The third cohort consisted of 265 mothers and children registered from January 1st to December 31st, 2016, in a paediatric practice in the city of Trieste [25, 26]. This corresponded to 18% of all the infants born in Trieste in 2016; the total number of live births in FVG in that year was 8,492. Data on breastfeeding were gathered during well-child visits at 1, 3, 5, 8, 12, 18, 24 and 36 months of age. The status of breastfeeding at discharge from the maternity ward was retrieved from hospital records. Apart from few missing or invalid data, the prevalence of any breastfeeding was available for 95% of the mothers at one month and 85% at 36 months of age.

In the three cohorts, data on breastfeeding were collected using the standard definitions and methods recommended by the WHO [27]. Before entering the data into the MMT, we set the initial sample size of each cohort as reference population. Then we entered into a spreadsheet numerators and denominators from the three cohorts, at each available age. After double checking for possible transcription mistakes, we copied the prevalence rates into the “country data” section of the MMT. When data for given ages (in months) were not available, the MMT added the missing data using its internal predictor. A breastfeeding rate chart, from birth to 36 months of age, was then generated, followed by several tables on actual and potential breastmilk production (litres), value estimates (USD), and lost breastmilk (litres and percentage). We repeated the same procedure for each cohort.

As far as the value estimates are concerned, the MMT assigns a monetary value of 100 USD per litre of breastmilk produced, based on the price of fresh human milk exchanged among human milk banks in Norway [17]. The MMT would allow to generate values in different currencies, by adding a rate of exchange. However, exchange rates vary over time. To avoid using different exchange rates and different currencies for each cohort study (Italian Liras in 1999 and EUR in 2007 and 2016), we decided to accept the MMT output in USD. Also, the exchange rates between USD and EUR fluctuate around 1. USD values, therefore, are a good proxy of EUR values.

Being based on published data that had been gathered after clearance by a committee of ethics, the study did not require to carry out further ethical clearance.

## Results

Table 2 shows the data on prevalence (%) of any breastfeeding at different ages (months) that were entered into the MMT from the three cohorts.

**Table 2:** Prevalence (%) of any breastfeeding by month of age in the three cohorts

Age in months	Cohort 1 (1999)	Cohort 2 (2007)	Cohort 3 (2016)
0 (at birth)	97% (793/818)	98% (388/396)	95% (240/252)
1	89% (739/830)	91% (261/287)	96% (242/252)
2	81% (669/826)		
3	73% (605/829)	82% (217/265)	89% (225/252)
4	67% (542/809)		
5	61% (483/791)		86% (223/252)
6	56% (447/798)	70% (165/235)	
7	50% (395/790)		
8	42% (338/805)		71% (163/231)
9	37% (295/798)	56% (97/173)	
10	31% (246/793)		
11	26% (204/784)		
12	21% (167/796)	39% (64/165)	59% (135/229)
18		20% (29/143)	35% (78/224)
24		12% (16/132)	25% (55/224)
36			7% (16/224)

While the rates of initiation of breastfeeding are similar in the three cohorts, it is clear that with time, from 1999 to 2016, the rates at subsequent ages increased. Improvements are observable from the first month and become large by age 5 to 6 months. At 12 months, the rate in the second cohort almost doubles the one of the first cohort, while the rate in the third cohort is almost three-fold higher. At 24 months, the rate in the third cohort is more than double the one of the second cohort.

**Fig. 1:**
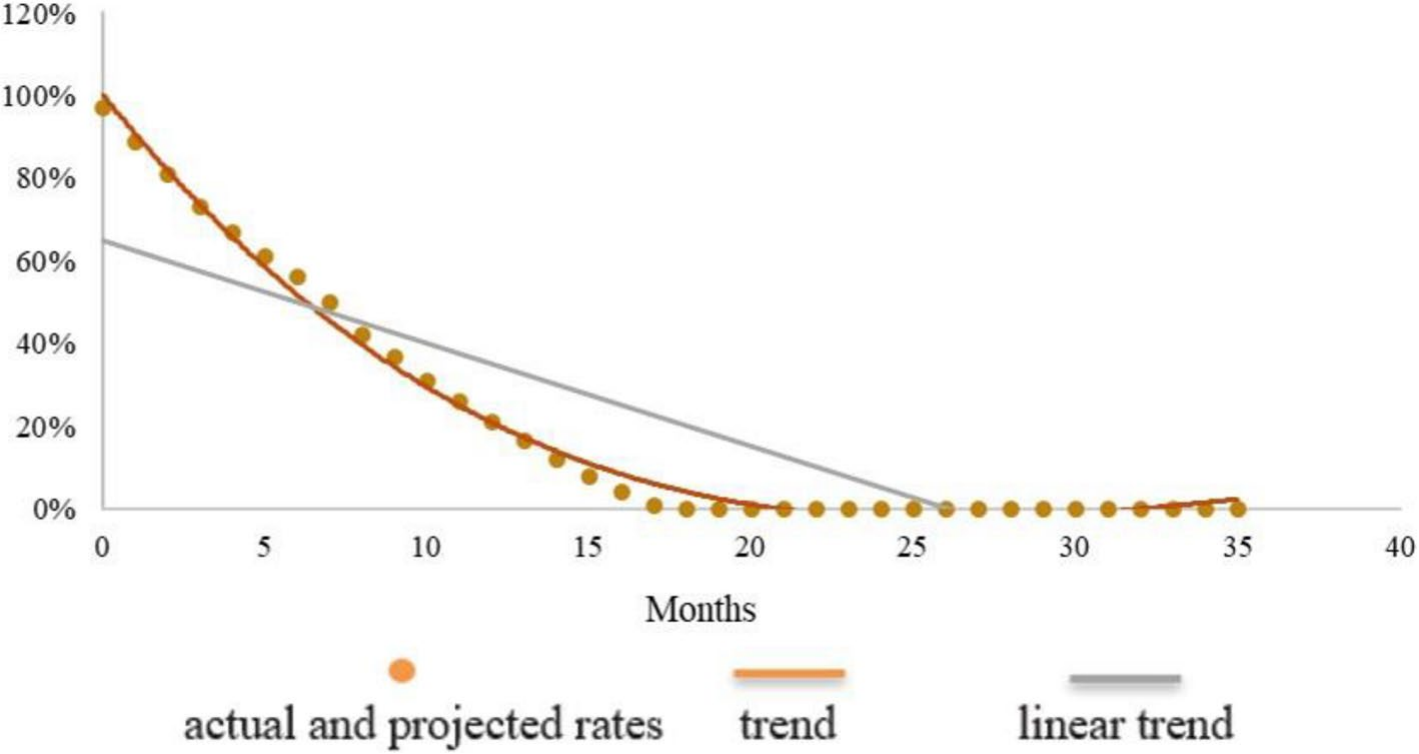
Cohort 1 (1999): actual and projected rates of any breastfeeding by month of age

**Fig. 2:**
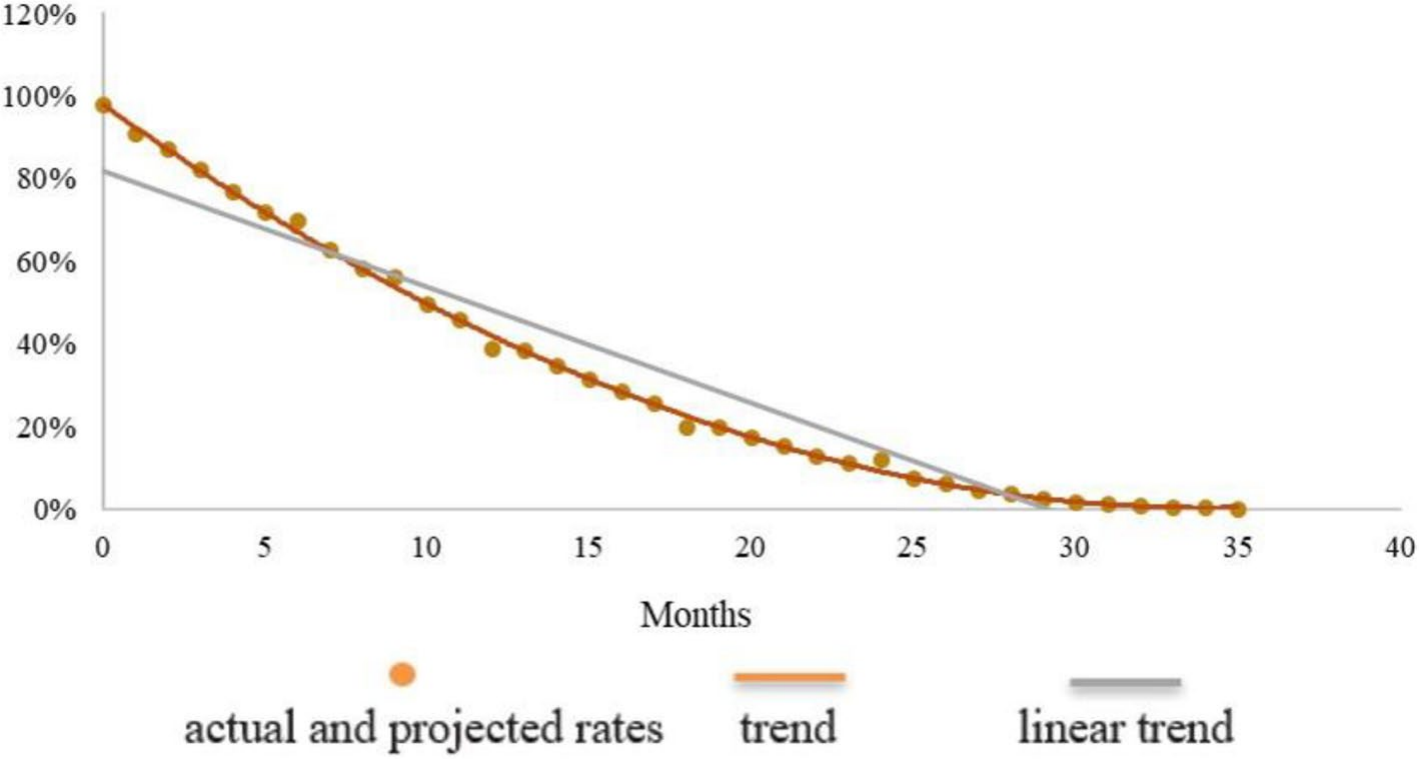
Cohort 2 (2007): actual and projected rates of any breastfeeding by month of age

**Fig. 3:**
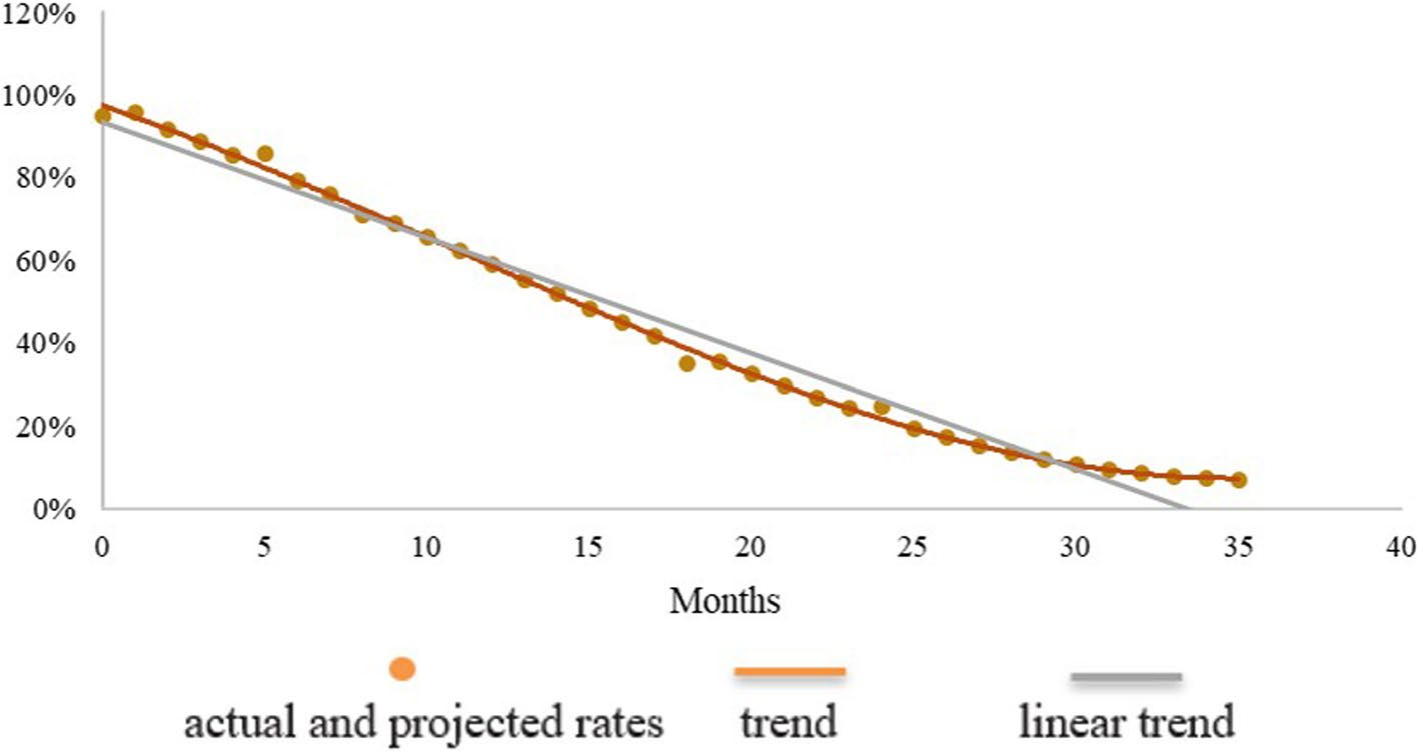
Cohort 3 (2016): actual and projected rates of any breastfeeding by month of age

Figures 1, 2 and 3 were created by the MMT and show the actual and projected rates of any breastfeeding in the first, second and third cohort, respectively.

The brown dots in the charts represent the data of Table 2, complemented by the data automatically added by the MMT predictor. In addition, a brown line is drawn by the MMT to smooth the curve, and a grey line is added to show the linear trend. In the third cohort (Fig. 3) this linear trend is almost laid over the dots of the prevalence rates; it slightly departs from the dots in the second cohort (Fig. 2), and it is even more divergent in the first one (Fig. 1). This is probably due to the fact that the 9 data entries of Fig. 3 are spread over three years of life, while the 13 data entries of Fig. 1 are concentrated in the first year of life (no data for the second and third year), and the 8 data entries of Fig. 2 stop at 24 months of age.

Table 3 shows the actual and potential production of breastmilk in the three cohorts, the volume and percentage of lost breastmilk, and the respective estimated value in million USD.

**Table 3:** Breastmilk production and value estimates in the three cohorts

	Cohort 1 (1999)		Cohort 2 (2007)		Cohort 3 (2016)	
	Volume (million litres)	Value (million USD)	Volume (million litres)	Value (million USD)	Volume (million litres)	Value (million USD)
Total for 3 years						
Actual production	0.11	10.85	0.08	7.13	0.06	5.66
Potential production	0.36	36.62	0.17	15.97	0.11	10.06
Lost breastmilk	0.24	22.77	0.09	8.84	0.05	4.40
Percent lost	67.7%		55.4%		43.7%	
Total 1st year						
Actual production	0.11	10.29	0.06	5.77	0.04	4.10
Potential production	0.17	16.44	0.08	7.81	0.05	4.92
Lost breastmilk	0.07	6.15	0.02	2.04	0.01	0.82
Percent lost	37%		26%		17%	

The top half of Table 3 considers any breastfeeding up to 36 months of age, the bottom half only the first year of life, when most breastmilk was produced. The improvement of the rates of any breastfeeding between 1999 and 2016 is reflected in the percentage of lost breastmilk: for children up to three years of age, more than two thirds in the first cohort, over a half in the second, and around 44% in the third. This means that from the 1999 to the 2016 cohort there was a 34% decrease in lost breastmilk. Considering only the first year of life, the percentage of lost breastmilk fell from 37% in the first cohort to 26% in the second and to 17% in the third; a 54% decrease.

With the above data, it is possible to estimate the volume and value of breastmilk for each mother and child dyad during the first 36 months of life. In the first cohort (*n* = 842), the average production of breastmilk per mother was about 130 L, for a value of about 13,000 USD per child. In the second cohort (*n* = 400), each mother produced on average 200 L of breastmilk and a value of 20,000 USD. In the third cohort (*n* = 265), the same estimates amount to 226 L and more than 22,600 USD. The increasing volumes and values by cohort reflect the improvements in breastfeeding rates.

## Discussion

This study shows that the MMT can be applied to datasets on breastfeeding at local levels, in addition to the national figures pre-loaded in the tool. The results refer to cohorts of children in different years in FVG; they should not be used to describe the current situation, nor generalised to Italy. Yet, they can be used to advocate with local and perhaps national health authorities, in Italy and elsewhere, for additional investments in the protection, promotion and support of breastfeeding. In FVG, such advocacy had already been exerted with the results of the 1999 cohort that had shown the amount of savings for the regional health system brought about by better breastfeeding rates: infants fully breastfed at three months, compared to those not or not fully breastfed, had lower cost of ambulatory (34.69 vs 54.59 EUR per infant/year) and hospital (133.53 vs 254.03 EUR per infant/year) care [20]. The advocacy was followed by investments in the training of health professional using the available WHO and UNICEF courses. More recently, when the regional monitoring system, active since 1998 [28], revealed that the trend of improvement of breastfeeding rates was slowing down and flattening, the regional health authority decided to finance a programme that introduced Problem Based Learning (PBL) as an alternative way to train health professionals on breastfeeding [29]. During and after the implementation of the PBL programme, the rates of breastfeeding started to increase again, probably helped also by the simultaneous introduction of biological nurturing as a strategy to improve the initiation of breastfeeding [30].

As already written, the increasing volumes and values of breastmilk from the first to the third cohort probably reflect improvements in breastfeeding rates over time. Although these are gross estimates and probably overestimates, because they are based on the initial number of subjects in each cohort and do not consider the loss to follow up, they are useful because they provide a sense of the mean volumes and values per mother and child dyad. These figures will be used for further regional advocacy and will hopefully lend even more support to the decision of the regional health authority to invest in breastfeeding. Overall, in the three cohorts, 1507 mothers produced around 250,000 L of breast milk. At 100 USD per litre, this would add up to around 25 million USD. During the years between 2000 and 2015, the average annual number of births in FVG was around 10,000. If the mothers of those infants had managed to breastfeed them at the same rate as the mothers in the three cohorts, an estimated 2 million litres of breastmilk would have been produced every year, with a value of around 200 million USD per year. The birth rate in the region has been decreasing since 2015 and the number of annual births has fallen under 8,000. The volume of breastmilk produced, however, may not have fallen proportionally, as the rates of breastfeeding are increasing.

Should data be available, it would be possible to use the MMT for advocacy at national level. As already mentioned, the more recent national figures date back to 2013 and refer only to initiation of breastfeeding and to exclusive and non-exclusive breastfeeding at 0–1, 2–3, 4–5 and 6–12 months of age [18]. The National Institute of Health piloted in 2018, and carried out in 2022, a surveillance system on child health that includes some results on breastfeeding for 19 out of 21 regions of Italy [31]. Data, however, refer only to exclusive breastfeeding at 2–3 (46.7%) and 4–5 (30%) months, to any breastfeeding at 12–15 months (36.2%), and to the percentage of children who were never breastfed (13%), with wide variation among regions and a downward north to south gradient. The MMT estimates the volume and value of breastmilk even with data at three time points. Ideally, more data would be needed to get more precise national estimates but, for the purpose of advocacy, even the 2022 surveillance data could be used for investments that may help reduce or close the gap between northern and southern regions.

Our study has obvious limitations. First, in the three cohorts there were losses to follow up, particularly large in the 2007 cohort. It is impossible to know if the reasons that lead many mothers in this cohort to withdraw from the study were somehow associated with breastfeeding; as a consequence, it is hard to decide whether the rates we used were over or under estimated. The loss to follow up was negligible in the other two cohorts, yet it leads to less precise estimates of the volumes and values of breastmilk per mother and child. Second, the rates of any breastfeeding for many months of age were missing and were therefore estimated by the MMT built-in predictor. This is reflected by the differences between actual and projected rates in the three Figures. This difference is considerable in the first cohort, from which data were available only up to 12 months of age, but less so in the second and third cohort. It is clear that complete monthly data would result in more precise estimates of volumes and values of breastmilk. Monthly data on breastfeeding, however, can hardly be gathered by routine data systems, but for advocacy purposes using quarterly data, for example, may not be a problem. Finally, for the estimates of the monetary value of breastmilk we used the 100 USD per litre of the MMT, as already written. Unfortunately, it is not possible to use a real local or Italian value. Although Italy has more than 40 human milk banks, there is no estimate of the commercial value of breastmilk because the Italian legislation does not allow its trade. There is, however, an estimate of the operational cost at a paediatric hospital in Rome in 2019: 231 euros per litre (771 L by 128 donors) [32]. The application of this value to our results would greatly increase the value of breastmilk produced and would lend further support to advocacy.

## Conclusions

The MMT was launched in May 2022. The downloadable version includes an advocacy page to which the results of the calculations can be attached. Yet, it is too early to rate it as an effective advocacy tool. Our study shows that, in addition to the country level, it can be used to estimate volumes and values of breastmilk produced and lost at local levels, where datasets on rates of any breastfeeding by month of age are available. It can also be used for before-and-after evaluations of interventions aimed at increasing the rates of breastfeeding. The challenge ahead is to start using the results to advocate for better and adequately funded programmes for the protection, promotion and support of breastfeeding. This may be relatively easy in FVG, but it will surely be more demanding at national level. More research is certainly needed to show how such a tool can be used to achieve results, including the incorporation of the monetary values of breastmilk and breastfeeding into national estimates of GDP.

## Data Availability

The datasets supporting the conclusions of this article are available from the corresponding author, with the exception of the data deriving from Cohort 2 (2007) which are in joint ownership with another academic institution.

## Abbreviations

AUD: Australian Dollar
EUR: Euro
FVG: Friuli Venezia Giulia
GDP: Gross Domestic Product
MMT: Mothers’ Milk Tool
PBL: Problem Based Learning
UNICEF: United Nations Children’s Fund
USD: United States Dollar
WHO: World Health Organization
